# Parenteral oestrogen in the treatment of prostate cancer: a systematic review

**DOI:** 10.1038/sj.bjc.6604230

**Published:** 2008-02-12

**Authors:** G Norman, M E Dean, R E Langley, Z C Hodges, G Ritchie, M K B Parmar, M R Sydes, P Abel, A J Eastwood

**Affiliations:** 1 Centre for Reviews and Dissemination, University of York, York YO10 5DD, UK; 2 Cancer Group, MRC Clinical Trials Unit, 222 Euston Road, London NW1 2DA, UK; 3 Hammersmith Campus, Department of Surgery, Imperial College Faculty of Medicine, London W12 0NN, UK

**Keywords:** parenteral oestrogen, prostate cancer, systematic review

## Abstract

The objectives of this study were to assess the effectiveness and safety of parenteral oestrogen in the treatment of prostate cancer, and to examine any dose relationship. A systematic review was undertaken. Electronic databases, published paper and internet resources were searched to locate published and unpublished studies with no restriction by language or publication date. Studies included were randomised controlled trials of parenteral oestrogen in patients with prostate cancer; other study designs were also included to examine dose–response. Study selection, appraisal, data extraction and quality assessment were performed by one reviewer and independently checked by another. Twenty trials were included in the review. The trials differed with regard to the included patients, formulation and dose of parenteral oestrogen, comparator used, outcome measures reported and the duration of follow-up. The results provide no evidence to suggest that parenteral oestrogen, in doses sufficient to produce castrate levels of testosterone, is less effective than luteinising hormone-releasing hormone (LHRH) or orchidectomy in controlling prostate cancer, or that it is consistently associated with an increase in cardiovascular mortality. Further well-conducted trials of parenteral oestrogen are required. A pilot randomised controlled trial comparing transdermal oestrogen to LHRH analogues in men with locally advanced or metastatic prostate cancer is underway in the United Kingdom.

Prostate cancer is now the most common male cancer, with nearly 35000 new cases in the United Kingdom in 2004 (Cancer Research UK, 2007). When prostate cancer is not amenable to potentially curative therapy, the aim of treatment is to reduce circulating levels of androgens and/or block them from initiating intracellular signalling. This can be achieved either surgically (by orchidectomy) or medically (referred to as androgen deprivation therapy (ADT), e.g., by luteinising hormone-releasing hormone (LHRH) analogues with or without anti-androgens).

Androgen deprivation therapy is now used extensively and for prolonged periods of time, and concerns are increasing about long-term toxicity, particularly osteoporosis. For example, a recent longitudinal study has shown that 35% of men who received LHRH therapy for prostate cancer will experience at least one skeletal fracture in the first 7 years and 19% will be diagnosed with osteoporosis/osteopaenia (Krupski *et al*, 2004). A number of clinical trials are underway to address whether adding a bone-strengthening agent, such as a bisphosphonate, to LHRH therapy would decrease the incidence of osteoporosis and other skeletal-related events. Several other side effects of castration, including hot flushes, anaemia, and metabolic syndrome, are also increasingly recognised, although others such as cognitive dysfunction require further research to clarify their impact (Sharifi *et al*, 2005). It has recently been suggested that the use of ADT is associated with earlier onset of fatal myocardial infarction in men aged 65 years or above (D'Amico *et al*, 2007) and that in patients with locoregional prostate cancer it may be associated with an increase risk of cardiovascular disease not seen with orchidectomy (Keating *et al*, 2006). An alternative approach is to evaluate agents that are potentially as effective as, or more effective than, LHRH analogues in activity, for example, by achieving castrate levels of testosterone while avoiding some of the side effects of the castration syndrome.

Oral oestrogen therapy, such as diethylstilboestrol (DES), has been used as a method of ADT and is reported to be as effective as surgical orchidectomy or LHRH analogues in producing castrate levels of testosterone (Byar, 1973). It also avoids some of the side effects associated with the castration syndrome such as osteoporosis, osteoporotic fractures and hot flushes. However, large randomised studies highlighted the cardiovascular system (CVS) toxicities associated with this form of therapy, which occurred in 30–35% of patients treated with the highest doses (Byar, 1973). The CVS side effects of oral oestrogen have been attributed to first-pass hepatic metabolism (von Schoultz *et al*, 1989). In contrast, parenteral oestrogen (e.g., administered intravenously, intramuscularly or transdermally) avoids first-pass metabolism in the hepatic circulation system and is not expected to be associated with the same incidence of CVS toxicity.

Two small pilot studies have been conducted evaluating oestrogen patches for patients with prostate cancer (Ockrim *et al*, 2003; Morganstein *et al*, 2004). Neither study reported any serious adverse events, and minor adverse effects such as skin irritation were insufficient to cause discontinuation of treatment. In light of concerns about osteoporosis from current methods of ADT and preliminary evidence that oestrogen patches might be a useful therapy, a systematic review was undertaken. The aim of this review was to assess the existing research on the use of parenteral oestrogen in prostate cancer, compared with alternative treatments, in order to inform the design of a randomised clinical trial comparing oestrogen patches with LHRH analogues (Abel, ongoing).

## MATERIALS AND METHODS

A systematic review protocol was developed with two principal aims: firstly, to investigate the clinical effectiveness and safety of parenteral oestrogen therapy in prostate cancer; and secondly, to examine any relationship between effectiveness or safety and oestrogen dose. Eighteen electronic databases, including MEDLINE, EMBASE and the Cochrane Central Register of Controlled Trials, were searched from inception to March 2004. Appropriate published paper and internet resources were also searched. (For a full list of sources searched and search terms used, see the full report by Dean *et al*, 2006.) The searches were updated in September 2007.

For the first review question, randomised controlled trials (RCTs) examining parenteral oestrogen in patients diagnosed with prostate cancer were included. Studies had to report at least one of the following outcome measures: overall survival, disease-free survival, disease progression, adverse events, quality of life, and economic costs. Inclusion criteria for the review question on dose–response were studies of any design that compared two or more doses of parenteral oestrogen in patients diagnosed with any stage of prostate cancer and reported at least one of the outcome measures listed above. Studies were assessed for inclusion and quality independently by two reviewers. The methodological quality of studies was evaluated independently using a modified form of the Jadad scale (Jadad *et al*, 1996). Data extraction was undertaken by one reviewer and independently checked by a second reviewer. All disagreements were resolved through consensus, and a third reviewer consulted where necessary. Multiple publications of a study were collated and extracted as a single study. No attempt was made to contact authors for missing data.

## RESULTS

### Nature of the evidence

**Figure 1:**
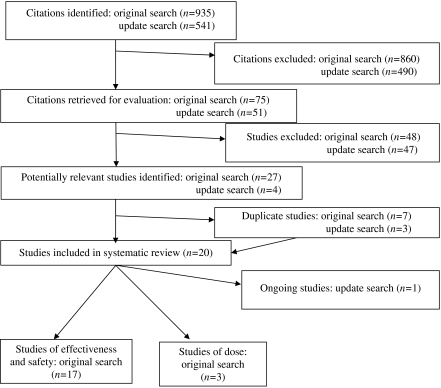
Study identification, retrieval and inclusion.

The literature search produced 935 citations, from which 75 potentially relevant published papers were obtained. After full assessment, 22 reports of 17 studies with a total of 3627 randomised patients were included in the review of effectiveness and safety, and three studies with a total of 82 patients in the review of dose (Figure 1). Full details of all the studies (including secondary outcomes not reported here) are available in the CRD report (Dean *et al*, 2006). There was considerable variation between the trials, which differed with regard to the profile of the patients included, the dose and formulation of parenteral oestrogen employed, the comparator used; the outcome measures reported, and the duration of follow-up.

The updated searches produced 541 citations (additional to those located in the original search), from which 51 additional potentially relevant published papers were obtained. After full assessment, three further reports of two previously included studies were identified in the review of effectiveness and safety (Mikkola *et al*, 2005, 2007; Hedlund *et al*, 2007). Two of these were full papers (Mikkola *et al*, 2005, 2007) and one was an abstract (Hedlund *et al*, 2007). In addition, an ongoing trial was identified that compares transdermal oestrogen administered through patches with an LHRH analogue, but for which no outcome data are available (Abel, ongoing). No further studies were identified in the review of dose.

#### Therapies and routes of administration

Of the 17 studies included in the review of effectiveness and safety, parenteral oestrogen was given alone in 9 studies with a total of 2192 patients (Jacobi *et al*, 1980; Steg *et al*, 1983; Bishop *et al*, 1989; Haapiainen *et al*, 1990; Aro *et al*, 1993; Lukkarinen and Kontturi, 1994; Mikkola *et al*, 1998; Henriksson *et al*, 1999; Hedlund *et al*, 2002), combined with oral oestrogen in 7 studies with a total of 1247 patients (Andersson *et al*, 1980; Daehlin *et al*, 1986; Haapiainen *et al*, 1986; Henriksson and Edhag, 1986; Aro *et al*, 1988; Johansson *et al*, 1991a; Lundgren *et al*, 1995), and combined with doxorubicin in 1 study with 188 patients (Leaf *et al*, 2003). Intramuscular (i.m.) polyoestradiol phosphate (PEP) was used in 14 of the 17 included studies (Andersson *et al*, 1980; Daehlin *et al*, 1986; Haapiainen *et al*, 1986, 1990; Henriksson and Edhag, 1986; Aro *et al*, 1988, 1993; Bishop *et al*, 1989; Johansson *et al*, 1991a; Lukkarinen and Kontturi, 1994; Lundgren *et al*, 1995; Mikkola *et al*, 1998; Henriksson *et al*, 1999; Hedlund *et al*, 2002), and 7 of the 9 studies using parenteral oestrogen alone (Bishop *et al*, 1989; Haapiainen *et al*, 1990; Aro *et al*, 1993; Lukkarinen and Kontturi, 1994; Mikkola *et al*, 1998; Henriksson *et al*, 1999; Hedlund *et al*, 2002). The other studies used intravenous (i.v.) Stilboestrol (Leaf *et al*, 2003), topical 17-*β*-diethyl-oestradiol (Steg *et al*, 1983) and i.m. oestradiol undecylate (Jacobi *et al*, 1980). The three studies included in the review of dose all used i.m. PEP alone (Henriksson *et al*, 1988; Stege *et al*, 1988, 1989).

Only one study used any transdermal administration, comparing transdermal 17-*β*-diethyl-oestradiol administered as a cream with oral Stilboestrol (Steg *et al*, 1983). This small study (*n*=42) was published more than 20 years ago and was poorly reported. The oestrogen dose of the topical ointment employed could not be accurately determined. It also used the surrogate outcome measure of urinary flow to assess tumour response. As this was the only identified evidence on the use of transdermal oestrogen, we focused on the higher quality evidence that was found on the use of i.m. PEP.

Pharmacokinetic studies suggest that a dose of at least 240 mg month^−1^ is required in order to produce sufficient oestradiol levels to rapidly suppress testosterone to castrate levels, in a manner similar to orchidectomy (Henriksson *et al*, 1999). Of the eight studies employing i.m. parenteral oestrogen alone, three used PEP doses of 240 mg month^−1^ (Mikkola *et al*, 1998; Henriksson *et al*, 1999; Hedlund *et al*, 2002), while the remainder employed 160 mg month^−1^ (Bishop *et al*, 1989; Haapiainen *et al*, 1990; Aro *et al*, 1993; Lukkarinen and Kontturi, 1994) or 100 mg month^−1^ (Jacobi *et al*, 1980). Seven studies combined PEP at 80 mg month^−1^ with oral ethinyl oestradiol at 150 *μ*g day^−1^ (Andersson *et al*, 1980; Daehlin *et al*, 1986; Haapiainen *et al*, 1986; Henriksson and Edhag, 1986; Aro *et al*, 1988; Johansson *et al*, 1991a; Lundgren *et al*, 1995), and the remaining study that assessed parenteral oestrogen in combination used 1 g Stilboestrol i.v. every 2 weeks with 50 mg m^−2^ doxorubicin every 3 weeks (Leaf *et al*, 2003).

#### Patient populations

There were differences between the trial participants in terms of their disease status, their prior treatment and their risk of CVS morbidity or mortality, including prior history of CVS complications. Some studies included only patients with metastatic disease, while others included only those with locally advanced prostate cancer. In some trials, there were stringent exclusion criteria for CVS history, while others did not exclude anyone on grounds of CVS health. Finally, some trials included only patients with newly diagnosed prostate cancer, while in others previous courses of non-hormonal treatment were permitted. Details of all the studies are presented elsewhere (Dean *et al*, 2006). Table 1 presents the study details for those studies which evaluated parenteral oestrogen alone at a dose of 240 mg month^−1^. It shows the variation in the patient populations between the studies, for example, all patients in the Hedlund trial had metastatic disease (Hedlund *et al*, 2002), compared with 45% in the Mikkola trial (Mikkola *et al*, 1998) and 12% in the Henriksson trial (Henriksson *et al*, 1999).

#### Study quality

None of the studies met all of the quality criteria; the majority of the studies were not reported in sufficient detail to allow full assessment of their methodological quality (Dean *et al*, 2006). In particular, the methods of randomisation and blinding were rarely described; only two reports contained enough detail to confirm that the method of randomisation was appropriate (Aro *et al*, 1993; Hedlund *et al*, 2002), one of which also reported that randomisation was concealed (Aro *et al*, 1993). Only one study reported that cardiovascular outcomes were assessed by a cardiologist blinded to the interventions (Hedlund *et al*, 2002).

### Trials using PEP at 240 mg month^−1^

In this report, emphasis is given to the three studies, including a total of 1394 patients, that used PEP at the biological minimally effective dose. The results of these studies are given in Table 2. The largest most recent study (*n*=917) was of good quality (Hedlund *et al*, 2002). This study randomised patients with T0-4 NX M1 prostate cancer to either PEP twice a month for 2 months and thereafter monthly, or to combined androgen blockade (flutamide and LHRH analogue or on an optional basis bilateral orchidectomy). A second study (*n*=444), by Mikkola *et al* (2007, 1998), randomised patients with T3-4 M0 or T1-4 M1 prostate cancer to either PEP or orchidectomy, and this study was of reasonable quality. The third study was a small phase II study, which randomised patients with newly diagnosed advanced prostate cancer to either PEP or orchidectomy (Henriksson *et al*, 1999).

#### Overall mortality

**Figure 2:**
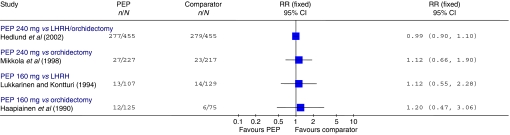
Overall mortality. Trials included in the figure are those where the parenteral oestrogen was PEP alone and for which data were fully reported. It should be noted that the follow-up times reported were not entirely uniform; where data for multiple time points were available those closest to 2 years are presented. Trials with zero events in any arm are not included in the figure.

**Figure 3:**
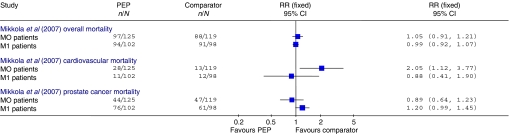
Overall, CVS and prostate cancer mortality at 10-year follow-up in the Mikkola trial for M0 and M1 subgroups. RRs shown are calculated from raw data presented in the publication and differ from those reported in the text, which were extracted from the Cox proportional hazards model reported.

The Hedlund study had high overall mortality (7 years after the start of the trial, with a median follow-up of 27 months, 61% of all patients had died) (Hedlund *et al*, 2002). There was no evidence of any difference in overall mortality between the treatment groups (RR=0.99, 95% CI: 0.90, 1.10; *P*=0.89) (Figure 2). This finding was confirmed by a brief report of a subsequent evaluation of data at 12-year follow-up, at which point 94% of patients had died (Hedlund *et al*, 2007). In the Mikkola trial, 76% of M0 and 93% of M1 patients had died at 10-year follow-up (Mikkola *et al*, 2007). The 2-year evaluation found no evidence of a difference in overall mortality between the treatment groups (RR=1.12, 95% CI: 0.66, 1.90; *P*=0.67) (Mikkola *et al*, 1998). Subgroup analysis using a Cox proportional hazards model at 10-year follow-up also showed no evidence of a difference between the PEP and the orchidectomy arm in either the M0 or the M1 patients (M0: RR=1.23, 95% CI: 0.92, 1.64; *P*=0.17; M1: RR=0.95, 95% CI: 0.70, 1.27; *P*=0.70) (Mikkola *et al*, 2007). The RRs calculated from raw data presented in the publication are shown in Figure 3. The Henriksson trial reported only one death in the orchidectomy group and none in the PEP group (Henriksson *et al*, 1999).

#### Prostate cancer mortality

**Figure 4:**
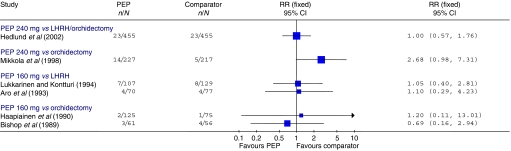
CVS mortality. Trials included in the figure are those where the parenteral oestrogen was PEP alone and for which data were fully reported. It should be noted that the follow-up times reported were not entirely uniform; where data for multiple time points were available those closest to 2 years are presented. Trials with zero events in any arm are not included in the figure.

The majority of mortality in the Hedlund study was reportedly due to prostate cancer; there was no evidence of any difference between treatment groups in the occurrence of cancer deaths (RR=0.95, 95% CI: 0.84, 1.07; *P*=0.39) (Hedlund *et al*, 2002) (Figure 4). This finding was confirmed by a brief report of a subsequent evaluation (Hedlund *et al*, 2007). There was also no evidence of a difference in prostate cancer mortality in the two arms of the Mikkola trial at 2 years (RR=1.09, 95% CI: 0.40, 2.96; *P*=0.86) (Mikkola *et al*, 1998); subsequent subgroup analysis using a Cox proportional hazards model at 10-year follow-up also showed no difference in disease-specific mortality in either M0 or M1 patients (M0: RR=1.14, 95% CI: 0.75, 1.73; *P*=0.55; M1: RR=1.07, 95% CI: 0.76, 1.50; *P*=0.72) (Mikkola *et al*, 2007). RRs calculated from raw data presented in the publication are shown in Figure 3. The small study by Henriksson *et al* (1999) reported no deaths due to prostate cancer.

#### CVS mortality

There was no evidence of a difference in CVS mortality in the Hedlund trial (RR=1.00, 95% CI: 0.57, 1.76; *P*=1.00) (Hedlund *et al*, 2002) (Figure 4). This finding was confirmed by a brief report of a subsequent evaluation (Hedlund *et al*, 2007). The Mikkola trial showed an increased level of CVS mortality in the PEP arm, although this was of borderline statistical significance (RR=2.68, 95% CI: 0.98, 7.31; *P*=0.05) (Mikkola *et al*, 1998). Subsequent subgroup analyses using a Cox proportional hazards model found this increase to be confined to M0 patients (RR=3.52, 95% CI: 1.65, 7.54; *P*=0.001); the subgroup with M1 disease showed no difference between the PEP and the orchidectomy groups (RR=0.92, 95% CI: 0.36, 2.36; *P*=0.86) (Mikkola *et al*, 2007). RRs calculated from raw data presented in the publication are shown in Figure 3. In this study, the authors retrospectively classified patients into those at high risk of CVS complications due to pretreatment vascular disease (patients with previous coronary heart disease, cerebral infarction, transient ischaemic attack or intermittent claudication), those with other pretreatment diseases associated with the risk of CVS events (patients with previous diabetes mellitus, hypertension, cardiac heart failure or rheumatoid arthritis), and those without any of the above pretreatment diseases (Mikkola *et al*, 2005). Within both the M0 and the M1 patient groups, the proportional hazards model found that the excess mortality in the PEP treatment arm was higher in those classified as having pretreatment vascular disease (M0: RR=3.48, 95% CI: 1.63, 7.44; *P*=0.001; M1: RR=3.13, 95% CI: 1.09, 9.00; *P*=0.035), but those with other pretreatment disease associated with CVS risk did not have significantly increased mortality (M0: RR=1.64, 95% CI: 0.59, 4.57; *P*=0.34; M1: RR=1.63, 95% CI: 0.40, 6.57; *P*=0.49) (Mikkola *et al*, 2007). There were no CVS deaths reported in the Henriksson trial (Henriksson *et al*, 1999).

#### CVS morbidity

**Figure 5:**
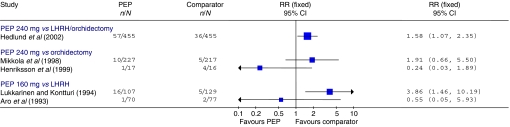
CVS morbidity. Trials included in the figure are those where the parenteral oestrogen was PEP alone and for which data were fully reported. It should be noted that the follow-up times reported were not entirely uniform; where data for multiple time points were available those closest to 2 years are presented. Trials with zero events in any arm are not included in the figure.

In contrast to the evidence on mortality, it appeared that CVS morbidity may occur at an increased rate in the PEP groups. In the Hedlund trial, this difference was statistically significant (RR=1.58, 95% CI: 1.07, 2.35; *P*=0.02) (Hedlund *et al*, 2002) (Figure 5). In particular, the incidence of both ischaemic heart disease (RR=3.40, 95% CI: 1.27, 9.14; *P*=0.02) and heart decompensation (RR=2.22, 95% CI: 1.02, 4.83; *P*=0.04) was significantly higher in the PEP arm. This finding was confirmed by a brief report of a subsequent evaluation (Hedlund *et al*, 2007). The Mikkola trial also initially reported higher levels of CVS morbidity in the PEP arm than the orchidectomy arm, but this difference was not statistically significant (RR=1.91, 95% CI: 0.66, 5.50; *P*=0.23) (Mikkola *et al*, 1998). However, subsequent subgroup analyses at 36-month follow-up revealed that there was a significantly greater incidence of CVS complications, including mortality, in the M0 patients (RR=3.40, 95% CI: 1.34, 8.59; *P*=0.01) but not in the M1 patients (RR=1.55, 95% CI: 0.61, 3.97; *P*=0.36) (Mikkola *et al*, 2005). As with CVS mortality, a Cox proportional hazards model found that the complication rate was significantly increased in those M0 patients with pretreatment vascular disease (RR=4.71, 95% CI: 1.69, 13.09; *P*=0.003), but not in those with other pretreatment disease associated with CVS risk (RR=2.25, 95% CI: 0.65, 7.83; *P*=0.20) (Mikkola *et al*, 2005). The Henriksson trial found more events in the orchidectomy group (4 out of 16) than in the PEP group (1 out of 17) (Henriksson *et al*, 1999).

### Trials using lower doses of parenteral oestrogen alone

Five trials used PEP or a similar preparation at 160 or 100 mg month^−1^. The results are shown in Table 2. There was no evidence of a statistically significant difference between treatment groups in either overall or prostate cancer mortality in the two studies that reported these outcomes. Cardiovascular mortality was reported by all five trials and did not differ significantly between treatment groups in any of the trials (*P*-values ranged from 0.29 to 0.91). Cardiovascular morbidity was reported in four trials (Jacobi *et al*, 1980; Bishop *et al*, 1989; Aro *et al*, 1993; Lukkarinen and Kontturi, 1994), and in only one, there was a statistically significant increase in the PEP group compared to the LHRH group (RR=3.86, 95% CI: 1.46, 10.19; *P*=0.01) (Lukkarinen and Kontturi, 1994).

### Trials using parenteral oestrogen combined with oral oestrogen or doxorubicin (PEP+)

Seven trials with a total of 1256 patients used PEP (i.m.) at 80 mg month^−1^ in combination with 150 *μ*g day^−1^ oral oestradiol (Andersson *et al*, 1980; Daehlin *et al*, 1986; Haapiainen *et al*, 1986; Henriksson and Edhag, 1986; Aro *et al*, 1988; Johansson *et al*, 1991a; Lundgren *et al*, 1995). One trial with 188 patients used Stilboestrol i.v. in combination with doxorubicin (Leaf *et al*, 2003). The results are given in Table 3. There was no evidence of a statistically significant difference between treatment groups in overall, prostate cancer or cardiovascular mortality in any of the studies that reported these outcomes. However, cardiovascular morbidity was higher in the PEP+ group than the comparator group for a number of studies; in particular, in one trial, recruitment to the PEP+ arm was terminated early due to the high number of cardiovascular events (Lundgren *et al*, 1995).

### Dose

Three studies with a total of 82 patients examined different doses of PEP (Henriksson *et al*, 1988; Stege *et al*, 1988, 1989). All were small and of uncertain or poor quality; one was a non-randomised trial (Henriksson *et al*, 1988). None of the studies reported mortality and in none of the studies any CVS events were reported (Table 4).

## DISCUSSION

This review of effectiveness and safety focused on the randomised evidence related to the use of i.m. PEP; only one small poor quality study of transdermal oestrogen cream was identified. The included trials were generally poorly reported or of poor quality. Nine studies evaluated parenteral oestrogen administered as monotherapy, and three of these used a clinically relevant dose of PEP (240 mg month^−1^), including two large recent RCTs, one of which was of good methodological quality. There was variation in the patient populations of these three studies, in particular, the proportion of patients with metastatic disease (all patients in the Hedlund trial had metastatic disease compared to less than half of patients in the Mikkola trial). The comparator arm was a mixed LHRH/orchidectomy arm in the Hedlund trial and orchidectomy alone in the other two trials. In addition, the analysis in the Mikkola trial was based on 10-year follow-up, although some data from 2-year follow-up were also included in the review. The Hedlund trial analysis was based on patients who had died within 7 years after the start of the trial with a median follow-up of 27 months; although data at 12-year follow-up have now been analysed, the report is currently only available in abstract form. A 24-month follow-up was reported in the Henriksson trial. Given the variation in study populations and outcomes, it was decided that it would not be meaningful to statistically pool these studies.

### Effectiveness

The randomised trials included in the review provided no clear evidence that parenteral oestrogen alone, at doses sufficient to produce castrate levels of testosterone, differed in effectiveness from LHRH analogues or orchidectomy in terms of prostate cancer mortality or overall mortality.

### Adverse events

The included studies also provided no consistent evidence that the incidence of fatal cardiovascular events with parenteral oestrogen alone is different to that reported with LHRH analogues or orchidectomy. Where there is evidence of excess mortality, the data suggest that it may be confined to those patients who have non-metastatic disease and to patients with pretreatment vascular disease; patients with other pretreatment disease associated with the risk of CVS events do not appear to incur a similar risk for excess mortality with PEP therapy (but the exclusion of, for example, hypertension and cardiac heart failure as high risk for CVS events may be considered contentious). However, there is clearer evidence of increased cardiovascular morbidity with parenteral oestrogen alone, although the seriousness of non-fatal adverse events was often not reported and criteria for recording an event were often unclear.

Such CVS risks should be balanced against the risks of non-CVS adverse events resulting from LHRH analogues or orchidectomy such as osteoporosis and osteoporotic fracture; these were reported in only one of the trials included in the review, which did show an increased rate of skeletal adverse events in the combined ADT arm (Hedlund *et al*, 2007). Other potential adverse events of LHRH analogues were not reported in any of the included studies. This lack of evidence on adverse events associated with standard hormone treatment may result partly from the relatively short follow-up times used in many of the studies, but also suggests that such events were not considered in the study design. Standard hormone treatment results in high levels of osteoporosis and, consequently, of osteoporotic fracture with a mortality rate of approximately 15% in the first year, although this has been reported to be as high as 35% (Center *et al*, 1999). Incidence of osteoporotic fracture should be an outcome measure for safety in any future trial.

Long-term follow-up is also of importance in risk assessment, given that there are indications that the effect of parenteral oestrogen on the incidence of CVS events may not be monotonic. For women on hormone replacement therapy, increased CVS risk over the first 2 years has been observed. Thereafter, CVS benefit accrues, such that the risk of CVS events is reduced by 30–50% after 10 years on treatment (Hulley *et al*, 1998; Grodstein *et al*, 2001). Similar changes may occur in men (Aro, 1991; Ockrim JL *et al*, 2006). This pattern of risk may result from changes in arterial compliance caused by oestrogen therapy. During the early months of oestrogen therapy, arterial compliance is reduced and cardiac demands increased. Thereafter, arterial compliance adapts leading to improved CVS dynamics (Turgeon *et al*, 2004). However, the Mikkola study examined the incidence of CVS events and found that the rate of occurrence did not diminish significantly over the first 3 years of treatment. Instead, the risk remained higher among men with non-metastatic disease in the PEP group than in the orchidectomy group, but did not differ between groups for those with metastatic disease (Mikkola *et al*, 2005).

### Costs

No economic evaluations were found. In addition to the cost of treatment regimes, the economic impact of the adverse events associated with alternative treatments requires evaluation. The annual cost of osteoporotic fractures in men in the United Kingdom has been estimated at £236 million (Pande and Francis, 2001). A previous economic evaluation of LHRH analogues has indicated that they are a cost-effective alternative to orchidectomy, but this is a hormonally equivalent treatment, which also results in the andropause (Aronson *et al*, 1999). A subsequent evaluation indicated that LHRH-based therapy was more cost-effective than orchidectomy or DES if initiated only after development of metastases (Bayoumi *et al*, 2000). One estimate suggests that hormonal therapy accounts for more than two-thirds of the total cost of prostate cancer, approximately £63.1 million for patients diagnosed during 2001–2002 in the United Kingdom (Sangar *et al*, 2005). These costs increase substantially with the addition of anti-androgens or bisphosphonates. In contrast, oestrogen patches cost approximately one-tenth the price of LHRH analogues (Ockrim J *et al*, 2006). Alternative transcutaneous administration using cream or gel is likely to be comparable in cost to that of patches.

## CONCLUSION AND FUTURE RESEARCH

Because of the paucity of good quality evidence, it is not possible to draw any clear conclusion on the effectiveness and safety of parenteral oestrogen compared with other hormone therapy. The results of this systematic review provide no evidence to suggest that parenteral oestrogen, in doses sufficient to produce castrate levels of testosterone, is less effective than LHRH or orchidectomy in controlling prostate cancer, or that it is associated with an increase in cardiovascular mortality. There is, therefore, a need for further research into the use of parenteral oestrogen as an alternative to existing hormone treatments for prostate cancer, which are associated with a number of side effects and high costs. Evaluation of parenteral oestrogen, involving direct comparison of the adverse event profile with that of LHRH analogues including, but not limited to, osteoporotic events, is needed. Particular attention should be paid to the issue of clearly defining and accurately assessing cardiovascular morbidity as well as mortality, especially in relation to a patient's existing cardiovascular disease status, particularly their history of vascular disease, and the metastatic status of their prostatic cancer. Patients' acceptability and quality of life issues should also be examined, along with a full economic evaluation. A pilot RCT of transcutaneous oestrogen patches *vs* LHRH analogues in prostate cancer is currently underway (Abel, ongoing). The primary objective of this study is to confirm that oestrogen patches are a safe and efficacious therapy for patients with locally advanced and metastatic prostate cancer.

## Figures and Tables

**Table 1:** Study details of trials employing PEP at 240 mg monthly

**Trial**	Hedlund *et al* (2002, 2007)	Mikkola *et al* (1998, 2005, 2007)	Henriksson *et al* (1999)
Study population	Advanced (T0–4, Nx, M1, grades 1–3)	Locally advanced (T3–4, M0) or metastatic (T1–4, M1)	Newly detected untreated advanced
Recruitment period	December 1992 to June 1997	January 1990 to March 1994	Not reported
Patients recruited (withdrawn)	917 (7)	444^a^ (not reported)	33 (0)
Disease status	T0 (*n*=5) 0.5%		
	T1 (*n*=33) 4%		
	T2 (*n*=146) 16%		
	T3 (*n*=493) 54%		T3 (*n*=22) 66%
	T4 (*n*=*n*=208) 23%		T4 (*n*=11) 33%
	(missing (*n*=25))		
	All M1 100%	T3–4, M0 (*n*=244) 55%	M0 (*n*=29) 88%
		T1–4, M1 (*n*=200) 45%	M1 (*n*=4) 12%
	G1 (*n*=136) 15%	G1 (*n*=111) 25%	
	G2 (*n*=414) 45%	G2 (*n*=261) 59%	G2 (*n*=20) 61%
	G3 (*n*=340) 40%	G3 (*n*=72) 16%	G3 (*n*=13) 39%
	(missing (*n*=20))		
Follow-up	Median follow-up:	2 years, subgroup analyses at 3 and 10 years	2 years
	PEP 27.1 months Comparator: 27.4 months, brief report for 12 years		
Dose	240 mg every 2 weeks for 2 months then 240 mg month^−1^	320 mg initial dose then 240 mg month^−1^	240 mg every 2 weeks for 2 months then 240 mg month^−1^
Comparator	Orchidectomy or combined androgen ablation	Orchidectomy	Orchidectomy
Adjuvant therapy	No	Pretreatment breast irradiation	Pretreatment breast irradiation
Cardiovascular criteria	No myocardial or cerebral infarct 1 month or less before the start of study	No symptomatic coronary heart disease	Patients with previous cardiovascular disease were included
Study quality	High	Randomisation poorly reported	Randomisation poorly reported

PEP=polyoestradiol phosphate.

a It is not clear whether this is the total number recruited to the study or whether it is the number of patients reported as being in the study.

**Table 2:** Included studies assessing parenteral oestrogen alone

**Study**	***N***	**Comparator**	**Follow-up**	**All-cause mortality**	**Prostate cancer mortality**	**Cardiovascular adverse events**	**Study quality**
*PEP at 240 mg monthly* a							
Hedlund *et al* (2002, 2007)	917	Comparator: triptorelin 3.75 mg month^−1^ i.m.+flutamide 250 mg t.i.d., p.o. (*n*=298) or optionally orchidectomy (*n*=159)	Median: PEP: 27.1 months Comparator: 27.4 months (brief report at 12 years)	PEP: 277/455 (60.9%) Comparator: 279/455 (61.3%)	PEP: 239/455 (52.5%) Comparator: 252/455 (55.4%)	PEP: 80/455 (17.6%), 23 fatal (5.1%) Comparator: 59/455 (13.0%), 23 fatal (5.1%)	High quality, blind outcome assessment, central randomisation
							
Mikkola *et al* (1998, 2005, 2007)	444	Orchidectomy	2 years; subgroup analyses at 3 and 10 years	2 years: PEP: 27/227 (11.9%)	2 years: PEP: 8/227 (3.5%)	2 years: PEP: 24/227 (10.6%), 14 fatal (7.5%)	Adequate study design but inadequate reporting of withdrawals
				Orchidectomy: 23/217 (10.6%)	Orchidectomy: 7/217 (3.2%)	Orchidectomy: 10/217 (4.6%), 5 fatal (2.3%)	
				10 years: M0: PEP: 97/125 (77.6%) Orchidectomy: 88/119 (73.9%) M1: PEP: 94/102 (92.2%) Orchidectomy: 91/98 (92.9%)	10 years: M0: PEP: 44/125 (35.2%) Orchidectomy: 47/119 (39.5%); M1: PEP: 76/102 (74.5%) Orchidectomy: 61/98 (62.2%)	Mortality at 10 years: M0: PEP: 28/125 (22.4%) Orchidectomy: 13/119 (10.9%); M1: PEP: 11/102 (10.8%) Orchidectomy: 12/98 (12.2%)	
							
Henriksson *et al* (1999)	33	Orchidectomy	2 years	PEP: 0/17 (0%) Orchidectomy: 1/16 (6.2%)	Not reported	PEP: 1/17 (5.9%) Orchidectomy: 4/16 (25.0%)	Pilot study, method of randomisation not described
							
*PEP at 160 mg monthly* a							
Lukkarinen and Kontturi (1994)	236	LHRH: goserelin s.c. depot injection 3.6 mg per 28 days	Mean: PEP: 23 months LHRH: 26 months	PEP: 13/107 (12.1%) LHRH: 14/129 (10.8%)	PEP: 3/107 (2.8%) LHRH: 3/129 (2.3%)	PEP: 23/107 (21.5%), 7 fatal (6.5%) LHRH: 13/129 (10.1%), 8 fatal (6.2%)	Inadequate reporting of withdrawals
							
Haapiainen *et al* (1990)	200	Orchidectomy	>2 years	PEP: 12/125 (9.6%) Orchidectomy: 6/75 (8.0%)	PEP: 6/125 (4.8%) Orchidectomy: 5/75 (6.7%)	CVS mortality: PEP: 2/125 (1.6%) Orchidectomy: 1/75 (1.3%)	Inadequate reporting of withdrawals
						Non-fatal events NR	
							
Aro *et al* (1988, 1989, 1993)	147	LHRH: buserelin 6.6 mg per 8 weeks; implant s.c.	3 years	NR	NR	PEP: 5/70 (7.1%), 4 fatal (5.7%) LHRH: 6/77 (7.8%), 4 fatal (5.2%)	Adequate study design
							
Bishop *et al* (1989)	117	Orchidectomy	NR	NR	NR	PEP: 8/61 (13.1%), 3 fatal (4.9%)	Insufficient information to assess
						Orchidectomy: 4/56 (7.1%), all fatal	
							
*Oestradiol undecylate at 100 mg monthly*							
Jacobi *et al* (1980)	42	Cyproterone acetate 300 mg week^−1^ i.m.	NR	NR	NR	PEP: 16/21 (76.2%), 2 fatal (9.5%)	Insufficient information to assess
						Cyproterone: 0/21 (0%)	
							
*5 mg β-diethyl-oestradiol applied as cream b.i.d.*							
Steg *et al* (1983)	56	Oral DES 1 mg t.i.d., p.o.	NR	NR	NR	Cream: 0/29 (0%) DES: 5/27 (18.5%), 2 fatal (7.4%)	Insufficient information to assess

CVS=cardiovascular system; LHRH=luteinising hormone-releasing hormone; *N*=number of patients; NR=not reported; PEP=polyoestradiol phosphate; PEP+=PEP combined with oral oestrogen.

Studies are ordered by sample size within dosage categories.

Since increased cardiovascular risk occurs primarily during the first 2 years of oestrogen therapy, where trials report CVS events for more than one follow-up period, those closest to 2 years are given.

a In some trials, participants may have had higher initial treatment doses or may have received other additional treatment. The dose given here is the routine dose given for the duration of the trial. Further details can be found in the full evidence tables (see Appendix 7 of CRD report).

**Table 3:** Included studies assessing parenteral oestrogen in combination with oral oestrogen or doxorubicin (PEP+)

**Study**	***N***	**Comparator**	**Follow-up**	**All-cause mortality**	**Prostate cancer mortality**	**Cardiovascular adverse events**	**Study quality**
*PEP at 80 mg monthly plus oral ethinyl oestradiol at 150 μg daily (PEP*+)^a^							
Lundgren *et al* (1995)	285	(1) Estramustine phosphate 280 mg b.i.d., p.o. (2) Surveillance, endocrine treatment on progression	⩾9 years	PEP+: 35/66 (53.0%) Estramustine: 40/74 (54.1%) Surveillance: 53/88 (60.2%)	PEP+: 8/66 (12.1%) Estramustine: 13/74 (17.6%) Surveillance: 25/88 (28.4%)	Events leading to withdrawal: PEP+: 37/66 (56.1%) Estramustine: 30/74 (40.5%) Surveillance: 11/88 (21.5%)	Large number of patients withdrawn and excluded from analysis. Recruitment to PEP+ arm terminated early due to high CVS event rate
							
Haapiainen *et al* (1986, 1991)	277	Orchidectomy	5 years	PEP+: 101/146 (69.1%) Orchidectomy: 86/131 (65.6%)	PEP+: 45/146 (30.8%) Orchidectomy: 47/131 (35.9%)	CVS mortality: PEP+: 35/146 (24.0%) Orchidectomy: 24/131 (18.3%)	Inappropriate randomisation (by date of birth)
							
Andersson *et al* (1980)	263	Estramustine phosphate 840 mg day^−1^ b.i.d., p.o.	⩾2 years	NR	NR	No significant difference between groups (values not reported)	Trial groups not clearly explained
							
Aro *et al* (1988)	151	(1) Orchidectomy (2) Radiotherapy 40 Gy (whole pelvis), 26 Gy (prostate) over 9 weeks including 3 weeks rest	4 years	PEP+: 16/50 (32.0%) Orchidectomy: 23/56 (41.1%) Radiotherapy: 9/45 (20.0%)	NR	PEP+: 18/50 (36.0%), 5 fatal (10%) Orchidectomy: 13/56 (23.2%), 6 fatal (10.7%) Radiotherapy: 6/45 (22.2%), 3 fatal (11.1%)	Inappropriate randomisation (date of birth)
							
Johansson *et al* (1991a, 1991b)	150	Orchidectomy	7–10 years (5 years for survival data)	PEP+: 54/74 (73.0%) Orchidectomy: 54/76 (71.1%)	PEP+: 27/74 (36.5%) Orchidectomy: 36/76 (47.4%)	PEP+: 36/74 (48.6%), 13 fatal (17.6%) Orchidectomy: 13/76 (17.1%), 9 fatal (11.8%)	Inappropriate randomisation (date of birth)
							
Henriksson and Edhag (1986); Henriksson and Johansson (1987)	91/100	Orchidectomy	⩾1 year	NR	NR	Major CVS events PEP+: 13/53 (24.5%) Orchidectomy: 0/47 (0%)	9 non-randomised patients included
							
Daehlin *et al* (1986)	30	(1) Estramustine phosphate 9.2 mg kg^−1^ day^−1^ b.i.d., p.o (2) Orchidectomy	6 months	NR	NR	PEP+: 1/10 (10%), 0 fatal (0%) Estramustine phosphate: 3/10 (30%), 1 fatal (10%) Orchidectomy: 0/10 (0%)	Insufficient information to assess
							
*1 g Stilboestrol i.v. every 2 weeks plus 50 mg m*^−*2*^ *doxorubicin every 3 weeks*^a^							
Leaf *et al* (2003)	188	Doxorubicin 50 mg m^−2^ every 3 weeks	>5 years	Median survival: PEP+: 8.5 months Doxorubicin: 7.7 months	NR	PEP+: 13.5%, 1.4% fatal Doxorubicin: 1.3%, 0% fatal	Insufficient information to assess

CVS=cardiovascular system; *N*=number of patients; NR=not reported; PEP=polyoestradiol phosphate; PEP+=PEP combined with oral oestrogen.

Studies are ordered by sample size within dosage categories.

Since increased cardiovascular risk occurs primarily during the first 2 years of oestrogen therapy, where trials report CVS events for more than one follow-up period, those closest to 2 years are given.

a In some trials, participants may have had higher initial treatment doses or may have received other additional treatment. The dose given here is the routine dose given for the duration of the trial. Further details can be found in the full evidence tables (see Appendix 7 of CRD report).

**Table 4:** Included studies comparing parenteral oestrogen given at different doses

**Study**	***N***	**Comparator**	**Follow-up**	**All-cause mortality**	**Prostate cancer mortality**	**Cardiovascular adverse events**	**Study quality**
*PEP at 320 mg* *monthly*							
Henriksson *et al* (1988)	38	PEP i.m.	Mean: 14.1 months	NR	NR	0 in all groups	Non-randomised trial
		(1) 240 mg month^−1^					
		(2) 160 mg month^−1^					
							
Stege *et al* (1988)	27	PEP i.m.	6 months	NR	NR	0 in all groups	Insufficient information to assess
		(1) 240 mg month^−1^					
		(2) 160 mg month^−1^					
							
*PEP at 320 mg* *monthly for 6 months reducing to 160 mg* *monthly for 6 months*							
Stege *et al* (1989)	17	PEP i.m. 320 mg month^−1^ for 6 months then 80 mg month^−1^ for 6 months	NR	NR	NR	0 in both arms	Insufficient information to assess

*N*=number of patients; NR=not reported; PEP=polyoestradiol phosphate.

Studies are ordered by sample size within dosage categories.
